# Increased sensitivity of African American triple negative breast cancer cells to nitric oxide-induced mitochondria-mediated apoptosis

**DOI:** 10.1186/s12885-016-2547-z

**Published:** 2016-07-29

**Authors:** Luis Martinez, Easter Thames, Jinna Kim, Gautam Chaudhuri, Rajan Singh, Shehla Pervin

**Affiliations:** 1 California State University, Dominguez Hills, Los Angeles, CA USA; 2 Columbia University New York, New York, NY 10027 USA; 3 Charles R. Drew University of Medicine and Science, Los Angeles, CA 90059 USA; 4 Department of Obstetrics and Gynecology, David Geffen School of Medicine at UCLA, Los Angeles, CA 90095 USA; 5 Jonsson Comprehensive Cancer Center at UCLA, Los Angeles, CA 90095 USA; 6 Division of Endocrinology and Metabolism, Charles R. Drew University of Medicine and Science, 1731 East 120th Street, Los Angeles, CA 90059 USA

**Keywords:** Breast cancer, Health disparity, African American, Unique biology

## Abstract

**Background:**

Breast cancer is a complex heterogeneous disease where many distinct subtypes are found. Younger African American (AA) women often present themselves with aggressive form of breast cancer with unique biology which is very difficult to treat. Better understanding the biology of AA breast tumors could lead to development of effective treatment strategies. Our previous studies indicate that AA but not Caucasian (CA) triple negative (TN) breast cancer cells were sensitive to nitrosative stress-induced cell death. In this study, we elucidate possible mechanisms that contribute to nitric oxide (NO)-induced apoptosis in AA TN breast cancer cells.

**Methods:**

Breast cancer cells were treated with various concentrations of long-acting NO donor, DETA-NONOate and cell viability was determined by trypan blue exclusion assay. Apoptosis was determined by TUNEL and caspase 3 activity as well as changes in mitochondrial membrane potential. Caspase 3 and Bax cleavage, levels of Cu/Zn superoxide dismutase (SOD) and Mn SOD was assessed by immunoblot analysis. Inhibition of Bax cleavage by Calpain inhibitor, and levels of reactive oxygen species (ROS) as well as SOD activity was measured in NO-induced apoptosis. In vitro and in vivo effect of NO treatment on mammary cancer stem cells (MCSCs) was assessed.

**Results and discussion:**

NO induced mitocondria-mediated apoptosis in all AA but not in CA TN breast cancer cells. We found significant TUNEL-positive cells, cleavage of Bax and caspase-3 activation as well as depolarization mitochondrial membrane potential only in AA TN breast cancer cells exposed to NO. Inhibition of Bax cleavage and quenching of ROS partially inhibited NO-induced apoptosis in AA TN cells. Increase in ROS coincided with reduction in SOD activity in AA TN breast cancer cells. Furthermore, NO treatment of AA TN breast cancer cells dramatically reduced aldehyde dehydrogenase1 (ALDH1) expressing MCSCs and xenograft formation but not in breast cancer cells from CA origin.

**Conclusions:**

Ethnic differences in breast tumors dictate a need for tailoring treatment options more suited to the unique biology of the disease.

## Background

Breast cancer is a complex disease where heterogeneous cell types contribute to its initiation and progression [1, 2]. It has been broadly classified into estrogen receptor positive (ER+) and estrogen receptor negative (ER-) sub types, each of which are dependent on specific environmental cues and signaling pathways for their development [3]. Frequent diagnosis of aggressive triple negative (TN) (ER-, progesterone receptor negative (PR-) and Her2 Neu-) form of breast cancer in young African American (AA) women suggest disparity in development of this deadly disease [4, 5]. Limited treatment options for these aggressive TN breast tumors causes high mortality rates in AA women [6]. In sharp contrast, Caucasian (CA) women are usually post-menopausal when they develop ER+ or TN breast tumors, which usually have better prognosis and lower mortality rates when compared to AA patients [7]. Even after adjustments for socio-economic factors, AA breast tumors appear to exhibit specific aggressive characteristics suggesting existence of unique biology contributed both by tumor cells and host microenvironment [8].

Using laser capture microdissection and genome-wide mRNA expression analysis, it has been reported that stroma of AA breast tumors had higher inflammation and angiogenesis when compared to similar tumors from the Caucasian populations [9]. In addition, several cell cycle regulators like p16, CCNA2, CCNB1 and CCNE2 as well as several biological processes including endoplasmic reticulum (ER)-associated degradation was found much higher in the tumor epithelium of AA than in the CA populations [9]. Ethnic differences were found in the increased expression of AMFR, a candidate oncogene that promotes metastasis, in AA when compared to CA breast tumors [9]. Furthermore, a tumor suppressor CDH13, was found hyper-methylated in AA when compared to CA breast tumors [10].

Our previous studies have indicated that AA and CA breast cancer cells respond differently to nitrosative stress, which is induced by nitric oxide (NO), a pleiotropic molecule that is produced by nitric oxide synthase (NOS) [11–13]. Oxidative/nitrosative stresses, which are produced by reactive oxygen species (ROS) /reactive nitrogen species (RNS) respectively influence all subtypes of breast cancer [14, 15]. Both endothelial nitric oxide synthase (eNOS) as well as inducible nitric oxide synthase (iNOS) have been detected in a large number of human breast tumors, where their expression patterns correlate with tumor grades [16]. However, ethnic differences in expression of NOS and response of AA and CA breast cancer cells to oxidative/nitrosative stress remains understudied. We have previously reported that AA TN breast cancer cell line, MDA-MB-468, was highly sensitive to NO-induced apoptosis [17]. NO was able to up regulate MAP kinase phosphatase (MKP-1) expression in MDA-MB-468 cells that promoted inactivation of ERK1/2, Bax integration into mitochondrial membrane leading to caspase-9 and -3 activation [17, 18]. However, NO was unable to increase MKP-1 expression or induce apoptosis in MDA-MB-231, a CA TN breast cancer cell line [17]. On the other hand, low (nM) concentrations of NO significantly up regulated proliferation of MDA-MB-231 cells by increasing translation of cyclin D1 and ornithine decarboxylase [19]. In this study, we have further examined responses of three additional TN breast cancer cell lines, from each of the ethnic populations, to nitrosative stress. Consistent with our previous studies, we found striking differences between AA and CA TN breast cancer cell lines towards nitrosative stress. NO specifically inactivated superoxide dismutase (SOD) to increase ROS that partly contributed to apoptosis in AA TN breast cancer cell lines. More importantly, NO treatment of AA breast cancer cell lines reduced mammary cancer stem cell (MCSC) content *in vitro* and attenuated xenograft formation *in vivo*. Our studies therefore, provide further evidence that there are ethnic differences in the biology of TN breast tumors and specific players in each population should be targeted for effective therapeutic interventions.

## Methods

### Materials

DETA-NONOate was purchased from Cayman Biochemicals (Ann Arbor, MI), Calpain Inhibitor III was from Calbiochem (Darmstadt, Germany), and N-acetyl-l-cysteine (NAC) was purchased from Sigma Aldrich (St. Louis, MO). Ac-DEVD-AMC was purchased from Pharmingen (San Diego, CA, USA). ApoAlert DNA Fragmentation Assay kit was obtained from Clontech (Mountain View, CA). SOD activity was measured by using an assay kit obtained from Cayman Chemical Company (Ann Arbor, MI). Aldetect Lipid Peroxidation Assay Kit was from Enzo Life Sciences (Ann Arbor, MI, USA). MitoTracker Red CMX-Ros dye was obtained from Life Technologies (Grand Island, NY).

### Human cell lines

All human breast cancer cell lines were obtained from American Type Culture Collection (ATCC) (Manassas, VA) in 2013. ATCC uses Promega PowerPlex 1.2 system and the Applied Biosystems Genotyper 2.0 software for analysis of amplicon. We have not done any further testing in our lab. MDA-MB-231, MDA-MB-157 and MDA-MB-436 breast cancer cell lines were propagated in Leibovitz’s L-15 medium containing 10 % FBS. HCC-1806, HCC-70, MDA-MB-468 and HCC-1395 were propagated in RPMI 1640 containing 10 % FBS. BT-549 was propagated in DMEM F-12 containing 10 % FBS.

### Cell viability

Cells seeded in six-well plate (7.5 × 10^5^ per well) were allowed to grow overnight. The cells treated with various concentrations of DETA-NONOate for 24 h were collected, and viability was determined by trypan blue exclusion method. The number of viable cells at each concentration and time point was determined in triplicate with a hemacytometer [20].

### TUNEL assay

The TUNEL assay was performed using ApoAlert DNA Fragmentation Assay kit from Clontech as described previously [21]. Briefly, cells (2×10^5^) were plated in 6 well plates, fixed in 1 % formaldehyde-PBS at 4 °C for 20 min. The cells were washed with PBS, and stored overnight in 70 % ethanol. The cells were treated with nucleotide mixture containing terminal deoxynucleotidyltransferase (Tdt) enzyme and incubated at 37 °C for 1 h. Cells were washed and analyzed under fluorescent microscope.

### Caspase-3 assay

Cells were lysed in insect cell lysis buffer {50 mm HEPES, 100 mM NaCl, 2 mM EDTA, 0.1 % 3-[(3-cholamidopropyl)dimethylammonio]-1-propanesulfonic acid (CHAPS), 10 % sucrose, 5 mM DTT, and 1× protease inhibitor} for 30 min at 4 °C. The lysates were used for caspase-3 (3 μg) assay using Ac-DEVD-AMC substrate, which after specific cleavage releases fluorescent AMC that was quantified using a fluorometer (Versa Fluro; Bio-Rad) with excitation at 380 nm and emission at 440 nm as described previously [22].

### Western analysis

For analysis of cytosolic proteins, cells were lysed in cell lysis buffer [50 mm HEPES (pH 7.5); 1 mm DTT, 150 mM NaCl, 1 mM EDTA, 0.1 % Tween 20, 10 % glycerol, 10 mm β-glycerophosphate, 1 mM NaF, 0.1 mm orthovanadate, 10 μg/ml leupeptin, 10 μg/ml aprotinin, and 0.1 mM PMSF] and were incubated at 4 °C for 30 min. Protein concentration was measured using Bio-Rad protein assay dye concentrate. Lysates (30 μg) were resolved electrophoretically on 10 % SDS-polyacrylamide gel and electrotransferred to a polyvinylidine difluoride membrane (Bio-Rad) using a tank blot procedure (Bio-Rad Mini Protean II). The membranes were incubated with the following primary antibodies: Heme oxygenase-1 (HO-1) (Santa Cruz, Cat # sc-10789), cleaved caspase-3 (Cell Signaling, Cat # 9661), Bax (Santa Cruz Biotechnologies, Cat # 20067), Bcl2 (BD-Transduction Laboratories, Cat # 551052), β-actin (Cell Signaling Technologies, Cat # 4967), NOX4 (Abcam, Cat # ab60940), Mn-SOD (Abcam, Cat # 13533), Cu/Zn SOD (Abcam, Cat #13498), COX IV (Abcam, ab14744) and 1:1000 dilutions of respective horseradish peroxidase-linked F(ab) fragment secondary antibody (Amersham Corp., Piscataway, NJ) for 1 h. Immunoreactive bands were visualized by enhanced chemiluminescence (ECL) detection system (Amersham) as described previously [20, 22].

### Measurement of MMP by flow cytometry

Cells (1X10^6^) were harvested after various treatments, washed twice with cold 1xPBS and incubated with 100 nM MitoTracker Red CMX-Ros dye at 37 °C for 15 min in the dark, washed twice with cold PBS, and analyzed immediately by flow cytometry, as described previously [20].

### Measurements of malondialdehyde and 4-hydroxy-alkenals

Levels of malondialdehyde and 4-hydroxy-alkenals (4-HAE) was measured using Aldetect Lipid Peroxidation Assay Kit (cat # BML-AK170-0001, Enzo Life Sciences, Ann Arbor, MI, USA) as per manufacturer’s instructions, described previously [23].

### Mitochondria and cytosolic cell fractionation

The cell fractionation was performed using mitochondria and cytoplasmic extraction reagents from Thermo Scientific (Rockford, IL, USA). The fractionation was done as described previously [17].

### Superoxide dismutase activity assay

Cells (1×10^6^) were seeded in six well plates to confluence and collected without use of proteolytic enzyme. SOD activity was measured by using an assay kit obtained from Cayman Chemical Company (Ann Arbor, MI). The activity assay was performed according to the manufacturer’s protocol.

### Aldefluor assay and flow cytometry

Aldefluor assay was carried out as described previously [24, 25] according to manufacturer’s (cat # 01700, Stem cell Technologies, Vancouver, Canada) guidelines. Briefly, breast cancer cells were suspended in Aldefluor assay buffer containing an ALDH substrate, bodipy-aminoacetaldehyde (BAAA) at 1.5 μM, and incubated for 40 min at 37 °C. To distinguish between ALDH^+^ and ALDH^−^ cells, a fraction of cells was incubated under identical condition in the presence of a 10-fold molar excess of the ALDH inhibitor, diethyl amino benzaldehyde (DEAB). This results in a significant decrease in the fluorescent intensity of ALDH^+^ cells and was used to compensate the flow cytometer. To determine CD44 expression, MDA-MB-231 and HCC1806 cells suspended in PBS were exposed to PE conjugated anti-human CD44 antibody (cat # 555479, BD Pharmingen™, CA, USA) and subjected to flow cytometry analysis.

### Xenograft formation

Six to eight week old nude mice (Harlan Laboratories Inc. Indianapolis, IN) were used for xenograft engraftment. Control or DETA-NONOate treated (24h) MDA-MB-468, HCC-70, HCC-1806 and MDA-MB-231 cells (2x10^6^ cells/100μl) were mixed with matrigel (1:1) and implanted subcutaneously (posterior dorsolateral) in the nude mice. Tumors were monitored over a period of 15 weeks and tumor volume was calculated as described previously [24, 26]. This study was carried out in strict accordance with the recommendations in the Guide for the Care and Use of Laboratory Animals of the National Institutes of Health. The protocol was approved by the Institutional Animal Care and Use Committee on the Ethics of Animal Experiments of the Charles R. Drew University of Medicine and Science (permit number: I-1103-261).

### Statistical analysis

Data are presented as mean ± S.D. and between-group differences were analyzed using ANOVA. If the overall ANOVA revealed significant differences, then pairwise comparisons between groups were performed by Newman–Keuls multiple comparison test. All comparisons were two-tailed, and *p*-values <0.05 were considered statistically significant. The experiments were repeated at least three times, and data from representative experiments are shown.

## Results

### Nitric Oxide preferentially induced cell death in AA TN breast cancer cells

**Fig. 1:**
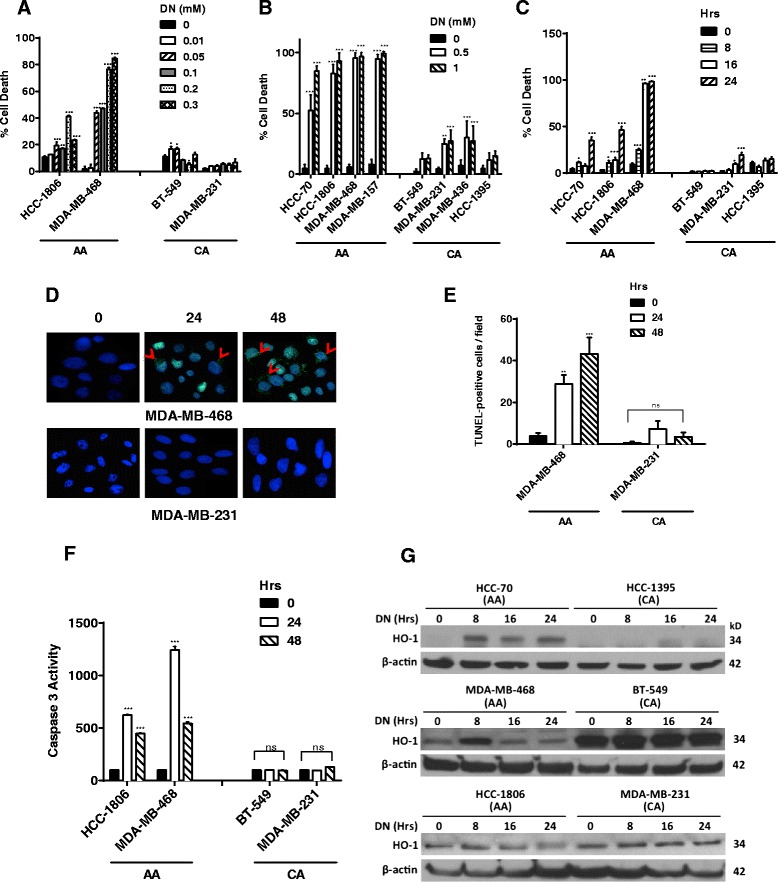
Nitric oxide preferentially induced cell death in AA breast cancer cells. AA and CA breast cancer cell lines were propagated in their respective media and treated with various concentrations of **a**) DETA-NONOate (DN)(0.01-0.3mM) for 48h. **b**) DETA-NONOate (0.5-1mM) for 48h **c**) DETA-NONOate (1mM) for 8-24h. Following these various treatments, the cells were harvested and their viability was determined by trypan blue exclusion method using hemocytometer for cell counting. **d**) Cells propagated on 8 well chamber slides were treated with DETA NONOate (1mM) for 24 and 48h. After these treatments the cells were fixed with 4% paraformaldehyde for 30 mins and DNA fragmentation determined by performing TUNEL assay. A representative picture of control and TUNEL positive MDA-MB-468 and MDA-MB-231 cells are shown. **e**) Quantitation of TUNEL positive cells was performed on images taken at 100 × magnification. Number of TUNEL positive cells/field (average of 3 fields) for each time points/cell line is shown. **f**) Cells treated with DETA-NONOate (1mM) for 48h were lysed in insect cell lysis buffer and 3μg of total cell lysates were subjected to caspase-3 activity assay using florescent substrates. Results from 3 different experiments were represented. **g**) Immunoblot analysis for heme oxygenase-1 (HO-1) was performed using 100μg of total cell lysates obtained from cells treated with DETA-NONOate (1mM) for various time points (0-24h). We used β-actin as housekeeping control. Two way ANOVA statistics was performed for A, B, C, E and F. Data are represented as mean ± SD with significant values presented as *p < 0.01, **p < 0.001, ***p < 0.0001

Effect of NO on viability and proliferation of breast cancer cell was examined by treatment with various concentrations of DETA NONOate, a long acting NO-donor. We analyzed four different breast cancer cell lines obtained from each of the ethnic populations. AA (HCC-1806, HCC-70, MDA-MB-157 and MDA-MB-468) and CA (BT-549, MDA-MB-436, HCC-1395 and MDA-MB-231) breast cancer cell lines were treated with various concentrations of DETA-NONOate for different time points. We and others have previously demonstrated that lower concentrations (1-100μM) of DETA-NONOate released physiological range (nM) while at higher concentrations (0.2-1mM) released high pathophysiological range (μM) of NO respectively [27, 28]. In this study, we observed that DETA-NONOate at 50μM induced (HCC-1806: 19.37 ± 2.58 %; MDA-MB-468: 43.94 ± 1.26 %); 100μM (HCC-1806: 17.21 ± 0.18 %; MDA-MB-468: 47.01 ± 0.36 %); 200μM (HCC-1806: 41.11 ± 0.89 %; MDA-MB-468: 76.46 ± 1.25 %); and 300μM (HCC-1806: 23.54 ± 0.27 %; MDA-MB-468: 84.78 ± 0.58 %) cell death at 48 h (Fig. 1a). In addition, high NO at 48 h. induced significantly higher cell death at 500μM (HCC-70: 52.10 ± 5.76 %; HCC-1806: 82.84 ± 3.36 %; MDA-MB-468: 95.35 ± 1.82 %; and MDA-MB-157: 94.71 ± 1.71 %) and at 1mM (HCC-70: 84.72 ± 1.83 %; HCC-1806: 92.80 ± 2.98 %; MDA-MB-468: 96.66 ± 1.57 %; and MDA-MB-157: 99.23 ± 0.769 %) (Fig. 1b). In all the four CA cell lines, no apparent cell death was observed at low concentrations of NO, while at high concentrations only 20-28 % cell death was observed at 48h in both MDA-MB-231 (27.07 ± 4.56 %) and MDA-MB-436 (27.19 ± 5.57 %) cancer cells (Fig. 1b). Even at 24h, 1mM DETA-NONOate was able to induce significant cell death selectively in AA TN breast cancer cells (HCC-70: 40.00 ± 3.99 %; HCC1806: 46.23 ± 3.42 %; and MDA-MB-468: 98.14 ± 0.66 %), while no significant cell death was observed in CA cells (Fig. 1c). We further performed terminal deoxynucleotidyl transferase dUTP nick end labeling (TUNEL) assay to assess whether DNA fragmentation occurred in TN AA breast cancer cells after 24 and 48 h of DETA-NONOate treatments. There was a significant increase in TUNEL-positive cells in AA but not in CA breast cancer cell line following DETA-NONOate (1mM) treatment (Fig. 1d-e). Quantitative analysis of TUNEL-positive cells in MDA-MB-468 cells showed significant increase at both 24h (28.71 ± 4.49 %) and 48h (43.27 ± 7.78 %) (Fig. 1e). No significant increase of TUNEL-positive cells were found in MDA-MB-231 cells at both time points following similar treatments (Fig. 1e). As DNA fragmentation is a hallmark of cells undergoing apoptosis where caspase-3 is the main executioner enzyme [29], we simultaneously examined caspase-3 activity in TUNEL-positive AA TN cells treated with NO. We found increased caspase-3 activity in AA TN cells (HCC-1806 and MDA-MB-468) with 1mM DETA-NONOate treatment as early as 24h, while no increase in activity was found in CA TN cells (Fig. 1f). Since the breast cancer cells from CA did not undergo apoptosis with NO treatment, we further examined the induction of HO-1, which is an early stress response marker [30]. We were able to detect early (8h after treatment) up-regulation of HO-1 protein levels in all the AA cell lines examined, while no significant change was found in CA TN breast cancer cells (Fig. 1g). These data indicate that NO increased cell death due to apoptosis in AA, while no detectable response was observed in CA TN breast cancer cells.

### NO induced mitochondria-mediated apoptosis in AA TN breast cancer cells

**Fig. 2:**
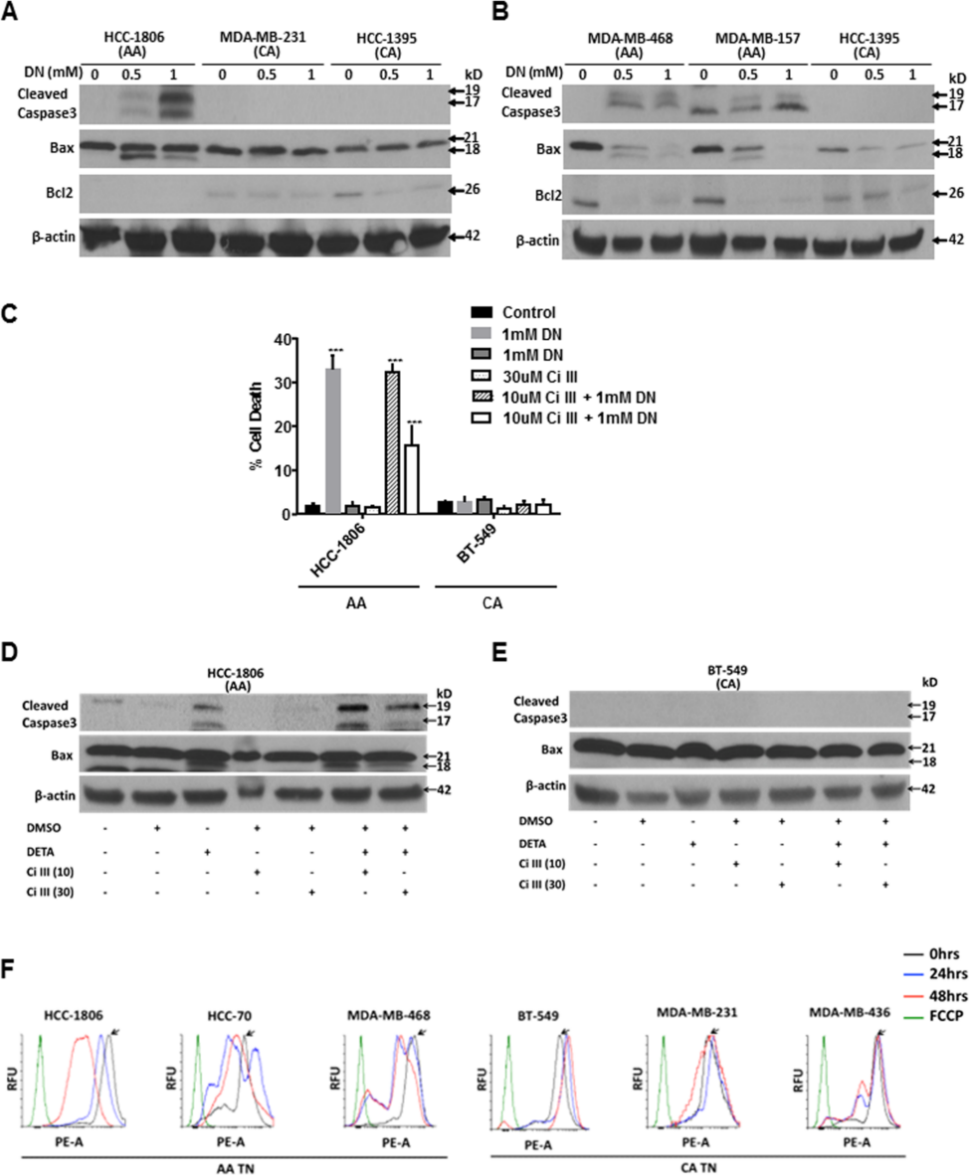
Nitric oxide induced mitochondria mediated apoptosis in AA breast cancer cells. **a**-**b**) Cells were seeded to confluence and treated with DETA NONOate (0.5-1mM) for 48h. Immunoblot analysis was performed for cleaved caspase-3 (19 and 17kDa band), total Bax (Intact Bax 20kD and cleaved Bax 18kD band) and Bcl2. Expression of β-actin was used as housekeeping control. Neither caspase-3 nor Bax cleavage was found in CA cell lines. **c**) Cells were exposed with DETA-NONOate (1mM) with or without pretreatment (1h) with various concentrations (10-30μM) of Calpain III (Ci III) inhibitor. Data is presented as mean ± SD with significant values presented as ***p < 0.001. **d**, **e**) Cells treated with DETA-NONOate and Calpain inhibitor III were subjected to Immunoblot analysis for cleaved caspase-3 and Bax. **f**) Cells treated with DETA NONOate for 24h (blue curve) or 48h (red curve) were harvested and incubated with Mito Tracker dye to measure mitochondrial membrane depolarization using flow cytometer. Uncoupling agent carbonyl cyanide-4-(trifloromethoxy) phenyl hydrazine (FCCP) was used as positive control (green curve) while untreated cells served as control (black curve)

We further examined whether mitochondria was involved in NO-induced apoptosis in AA TN breast cancer cells. Integrity of mitochondrial membrane, which is maintained by the ratio of pro- apoptotic and anti-apoptotic proteins, releases cytochrome *C* when compromised to activate caspase-3 [31]. Cleaved Bax, a pro-apoptotic molecule, has been found to integrate into the mitochondrial membrane to release cytochrome *C* [17]. Cleaved caspase-3 as well as cleaved Bax, which is an active form of Bax, was detected in all AA but not in CA TN breast cancer cells with 48h of DETA-NONOate (1mM) treatment (Fig. 2a-b). Interestingly, basal levels of Bcl2, an anti-apoptotic molecule was undetectable in HCC-1806, while its levels dramatically declined in MDA-MB-468 and MDA-MB-157 AA TN breast cancer cells with NO treatment (Fig. 2a-b). In sharp contrast, significant Bcl2 levels were detected in all CA TN breast cancer cells and remained relatively stable or even slightly increased upon NO exposure (Fig. 2a-b). Since Bax cleavage was a common occurrence in all AA TN cells undergoing apoptosis, we inhibited Bax cleavage to examine its contribution to NO-induced apoptosis. Calpain, which gets activated by oxidative stress, cleaves Bax at the N*-*terminal to generate a potent pro-apoptotic 18-kDa fragment that promotes Bcl-2-independent cytochrome *C* release and apoptotic cell death [32]. We found that pre-treatment with Calpain inhibitor III caused 52.74 ± 4.56 % reduction in cell death with NO treatment in HCC-1806 AA TN breast cancer cells as assessed by trypan blue exclusion assay (Fig. 2c). No change in cell viability was observed in NO treated CA breast cancer cells with or without Calpain inhibitor III. Immunoblot analysis shows that in addition to reduction in cell death, there was reduced Bax and caspase-3 cleavage with Calpain inhibitor III treatment in AA but not in CA breast cancer cells (Fig. 2d-e).

Apoptotic stimuli initiates a series of changes in the mitochondria that are crucial to the death program [33, 34]. One of the changes is opening of large pores in the mitochondrial membrane leading to mitochondrial permeability transition (PT) and disruption in the mitochondrial membrane potential (MMP), which are early obligatory step in the death program [35]. We, further examined changes in MMP in AA and CA TN breast cancer cells with NO treatment. We found AA TN breast cancer cells upon NO exposure underwent significant depolarization of MMP as early as 24h with further increase at 48h (Fig. 2f). On the contrary, with NO treatment, there was hyperpolarization of mitochondrial membranes in CA breast cancer cell lines (Fig. 2f). These data indicate that NO induced mitochondria-mediated apoptosis specifically in AA but not CA TN breast cancer cells.

### NO increased oxidative stress to induce mitochondria-mediated apoptosis in AA TN breast cancer cells

**Fig. 3:**
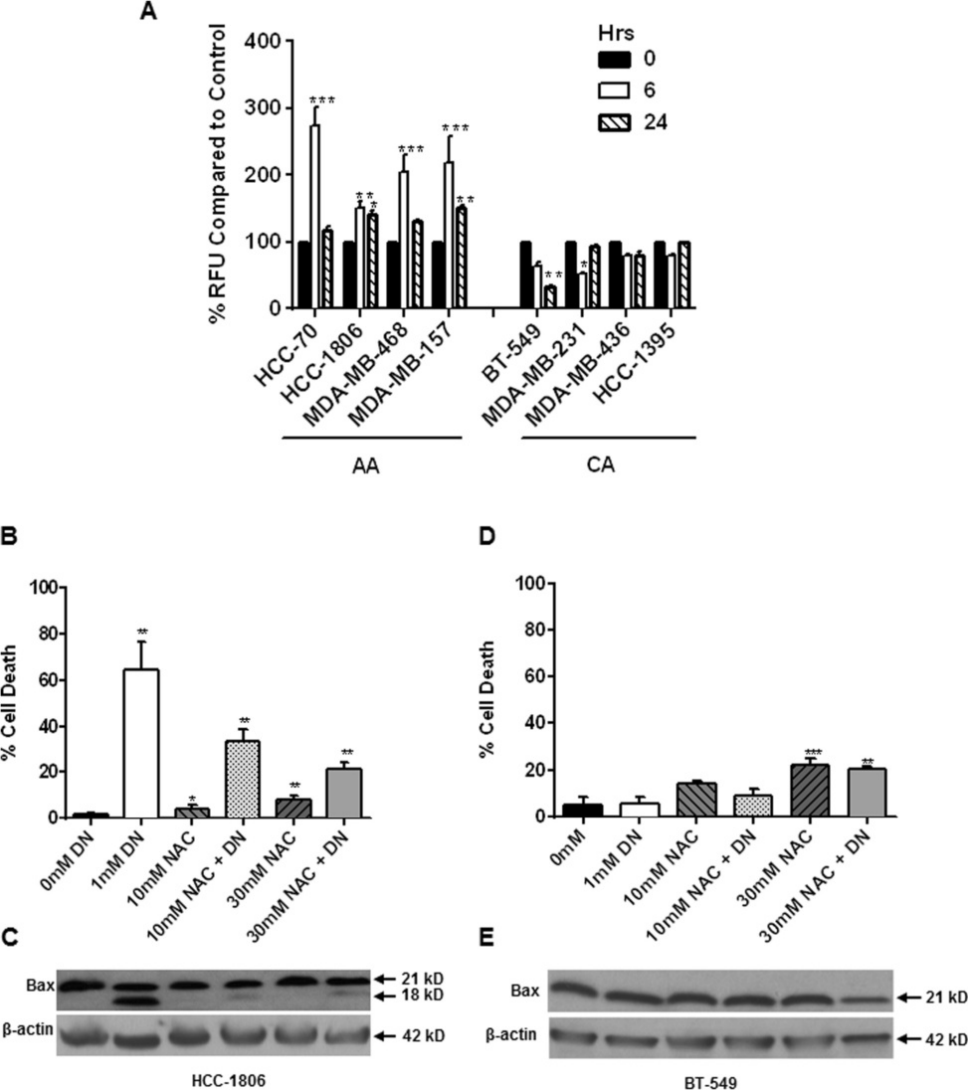
Nitric oxide–induced apoptosis in AA breast cancer cells was dependent on increase in ROS. **a**) Cells seeded in 96 well plates were treated with DETA NONOate (1mM) for different time points (6-24h). 4-hydroxy-alkenal levels was measured using Aldetect Lipid Peroxidation Assay kit as per manufacturer’s instruction. Cells were seeded to confluence and treated with DETA NONOate (1mM) for 24h with or without pretreatment (1h) with N acetyl-cysteine (NAC) at various concentrations (10-30mM). **b**, **d**) Cell viability was measured by trypan blue exclusion method using hemocytometer for cell counting. **c, e)** Immunoblot analysis of total cell lysates was performed for Bax (lower panels). Data are represented in Mean ± SD with significant values presented as *p < 0.01 and **p < 0.001

NO has been found to react with ROS, more specifically with superoxide (O_2_^-^) , to generate peroxynitrite and oxidative/nitrosative stress that could contribute to depolarization of MMP and mitochondria-mediated apoptosis [28]. We measured malondialdehyde and 4-hydroxyalkenals, which have been used as an indicator of lipid peroxidation and oxidative stress, in NO treated cells. Concentrated lysates of control and NO treated cells were subjected to colorimetric assay to detect malondialdehyde and 4-hydroxyalkenals using kit and manufacturer’s instructions as mentioned in Materials and Method. There was increase in oxidative stress with DETA-NONOate (1mM) treatment as early as 6h in HCC-70 (273.79 ± 27.27 %), HCC-1806 (151.04 ± 9.38 %), MDA-MB-468 (204.20 ± 25.26 %), and MDA-MB-157 (218.87 ± 40.07 %) AA TN breast cancer cells (Fig. 3a). No significant increase in oxidative stress was detected in any of the CA TN breast cancer cells examined (Fig. 3a). We further pre-treated AA breast cancer cells with N-acetyl cysteine (NAC), a ROS quencher, to determine whether increase in ROS with NO treatment contributed to apoptosis. Pre-treatment of cells with NAC significantly reduced NO-mediated cell death in HCC-1806 (67.13 ± 1.57 %) (Fig. 3b). Immunoblot analysis showed that NO-induced high Bax cleavage in HCC-1806 cells was attenuated by pretreatment with NAC (Fig. 3c). On the contrary in CA TN cell lines, NAC treatment led to slight increase in cell death in a concentration-dependent manner (Fig. 3d). No significant cleavage of Bax was observed in CA breast cancer cells with any of the treatments (Fig. 3e). These data indicate that increased levels of ROS in NO treated AA TN breast cancer may contribute to the observed cell death.

### NO inactivated SOD to increase ROS only in AA TN breast cancer cells

**Fig. 4:**
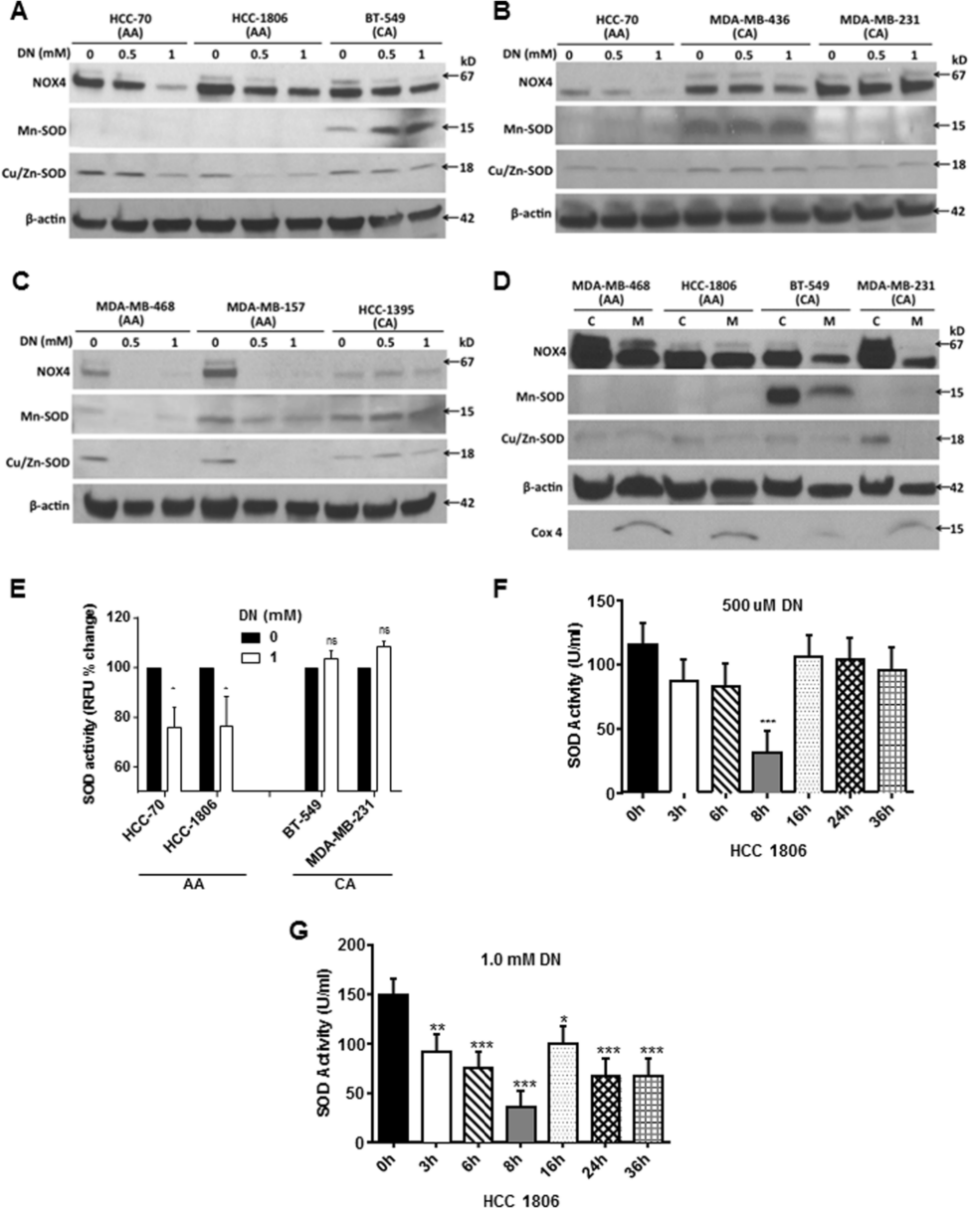
Nitric oxide inactivates SOD to increase ROS in AA TN breast cancer cells. **a**-**c**) Cells treated with DETA-NONOate (0.5 mM and 1mM) for 48h was subjected to immunoblot blot analysis for NOX4, Mn-SOD and Cu/Zn SOD where β-actin was used as control. **d**) Control and DETA NONOate (1mM) treated cells were subjected to cell fractionation using kit to separate cytoplasmic and mitochondrial fraction as per manufacturer’s instructions. These fractions were further subjected to immunoblot blot analysis for NOX4, Mn-SOD, and Cu/Zn SOD. COX4 was used as positive control for purity of mitochondrial fraction. **e**, **f**, **g**) cells treated with DETA-NONOate (0.5 mM and 1mM) for various time points (3-36h) were subjected to SOD activity assay using kit as per manufacturer’s instructions. Data are represented in Mean ± SD with significant values presented as *p < 0.01 and **p < 0.001

**Fig. 5:**
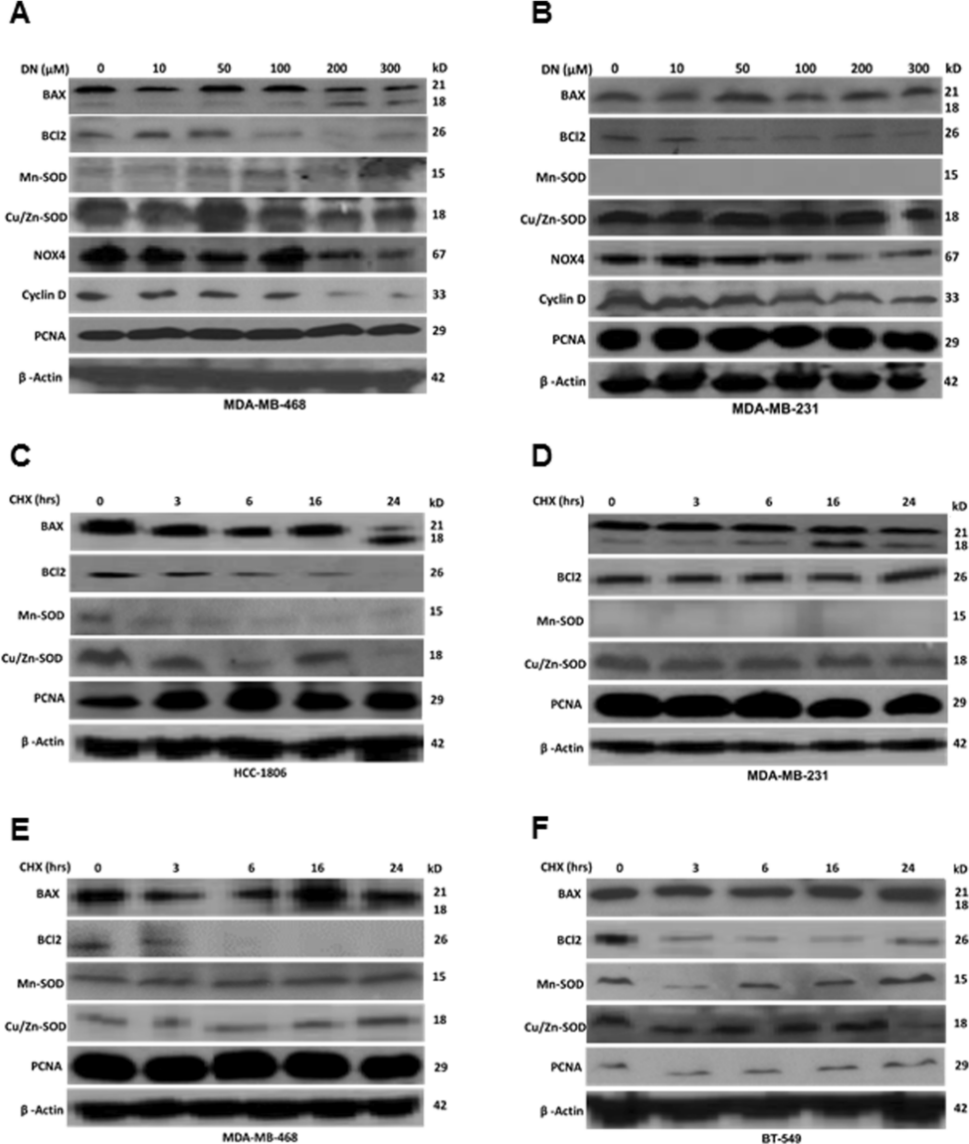
**a**-**b**) Cell lines (AA and CA TN cells) were treated with various concentrations (1-300μM) of DETA NONOate for 24h after which they were harvested, lysed and subjected to immunoblot analysis for BAX, Bcl2, Mn-SOD, Cu/Zn SOD, NOX4, cyclin D, and PCNA. β-actin was used as control. Experiments were repeated at least three and a representative data is shown. **c**-**f**) Cells treated with cycloheximide (CHX) for various concentrations were subjected to immunoblot analysis for BAX, Bcl2, Mn-SOD, Cu/Zn SOD, NOX4, cyclin D, and PCNA. β-actin was used as control

Since NO exposure was able to increase ROS levels in AA TN breast cancer cells, we further examined the levels of mitochondrial manganese superoxide dismutase (Mn-SOD) and cytosolic copper/zinc SOD (Cu/Zn-SOD). These metalloenzymes are most effective intracellular enzymatic antioxidants, known to hydrolyze ROS to H2O2, which further dissociates to O_2_ and H_2_O [36]. Levels of both SODs were examined after treatment with DETA-NONOate (0.5-1mM) for 24 and 48h. No significant decline in the levels of Mn-SOD or Cu/Zn SOD was observed in either AA or CA breast cancer cells at 24h of DETA-NONOate treatment (data not shown). However, at 48h, DETA-NONOate (0.5-1mM) caused a dramatic reduction in the levels of both Mn-SOD and Cu/Zn-SOD in AA but not in CA breast cancer cells (Fig. 4a-c). On the contrary, in some CA cell lines there was an increase in the levels of Mn-SOD (BT-549 and HCC-1395), while in another cell line (MDA-MB-436) there was no significant change from control with NO treatment (Fig. 4a-c). With cell fractionation, we found considerable amount of Mn-SOD in the mitochondria of CA (BT-549) but not in AA (HCC-70 and HCC-1806) breast cancer cells (Fig. 4d). We did not find any difference in levels of NOX4 protein, which is known to produce ROS, in AA and CA breast cancer cells (Fig. 4a-d). The purity of mitochondria was assessed by examining COX IV protein levels in the mitochondrial fraction (Fig. 4d). Since reduction in the protein levels of SOD at later time points of NO treatment did not coincide with early increase in ROS, we further examined the SOD activity in these cells. We found exposure to NO (DETA NONOate 1mM, 48h) reduced SOD activity (HCC-70: 19.15 ± 7.81 %; HCC-1806: 17.22 ± 6.51 %) in AA breast cancer cells (Fig. 4e). In sharp contrast to decline in AA breast cancer cells, there was some increase in SOD activity in all the CA TN breast cancer cells with NO treatment (BT-549: 3.80 ± 2.87 %; MDA-MB-231: 8.63 ± 2.06) (Fig. 4e). We further examined SOD activity after shorter exposures to various concentrations of NO in an AA TN breast cancer cell line (HCC-1806). With lower levels of NO (DETA-NONOate 500μM) we found a decline in SOD activity as early as 3h (25.01 ± 9.94 %), which continued to reduce till 8h (72.83 ± 7.73 %) after which it returned to near control by 16-24h (Fig. 4f). With higher NO (DETA-NONOate 1mM), the SOD activity continued to decline till 8h (76.91 ± 5.2) after which it remained lower than the control (Fig. 4g). Since lower concentrations of NO reduced proliferation of AA breast cancer cell lines, we further examined its effect on Mn SOD and Cu/Zn SOD as well as on various proliferation (cyclin D1 and PCNA), apoptosis (Bax and Bcl2), and oxidative stress (NOX4) markers. We did not find changes in the levels of Mn SOD or Cu/Zn SOD in any of the cell lines examined, while the levels of cyclin D1 declined in MDA-MB-468 cells treated with lower concentrations of NO (Fig. 5a, b). In addition, while no significant changes in Bax and Bcl2 was found, there was some decline in NOX4 protein levels in both the cell lines examined. We further examined the stability of these proteins after treating the cells with non-specific protein synthesis inhibitor, cycloheximide, for different time points. No significant changes in the levels of Cu/Zn SOD was observed in MDA-MB-468, MDA-MB-231 and BT-549 breast cancer cells, while in HCC-1806 cells, there was an initial decline followed by an increase at later time points of cycloheximide treatment (Fig. 5c-f). In addition, no significant differences in the levels of Mn-SOD with cycloheximide treatment was detected. However the levels of Bcl2 rapidly declined and remained lower in AA when compared to CA breast cancer cells treated with cycloheximide (Fig. 5c-f). Levels of Bax however remained unchanged in these cells while its cleavage increased at later time points of cycloheximide treatment (Fig. 5c-f). Levels of proliferating cell nuclear antigen (PCNA) remained constant in all the cell lines with cycloheximide treatment (Fig. 5c-f). The above data shows that NO inactivates SOD to increase oxidative stress specifically in AA but not CA TN breast cancer cells. In addition, there was no significant difference in the stability of Mn-SOD or CU/Zn-SOD in the AA and CA TN breast cancer cells.

### NO treatment reduced Mammary Cancer Stem Cell content in AA breast cancer cells

**Fig. 6:**
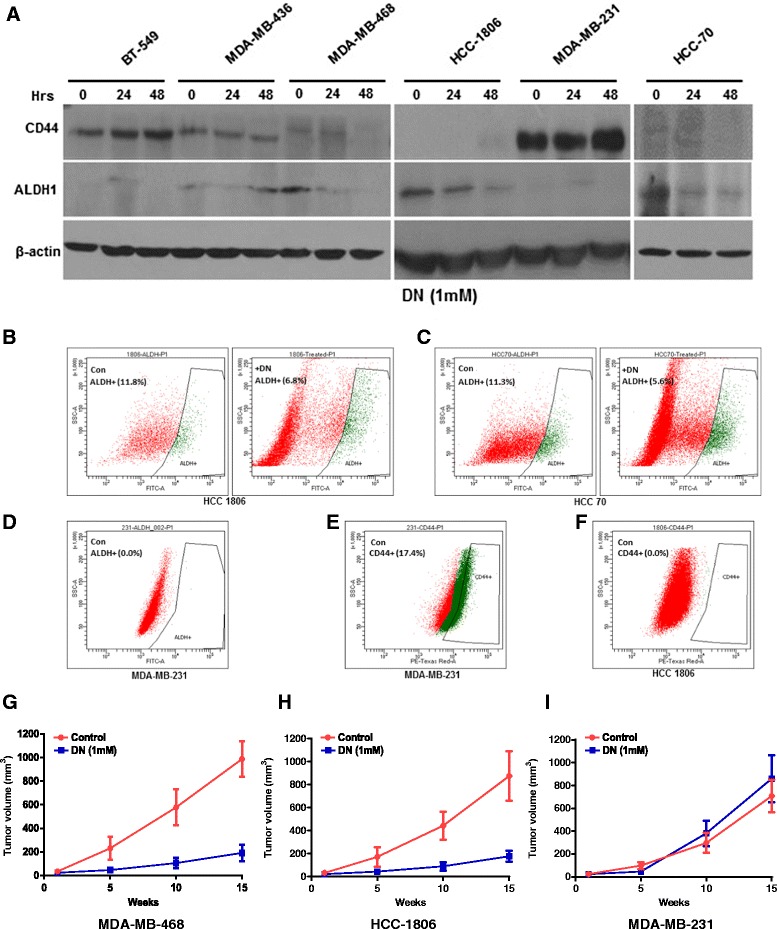
**a**) Breast cancer cell lines treated with DETA NONOate (1mM) for 24 and 48h were examined for MCSC markers ALDH1 and CD44 by Immunoblot analysis. **b**-**c**) Breast cancer cell lines, HCC1806 and HCC70 were treated with DETA NONOate (1mM) for 24-36h. Control and treated cells were suspended in ALDH1 assay buffer and subjected to ALDH1 activity assay using florescence activated cytometric analysis. Both dead and live populations in control and treated cells were gated to detect ALDH1 activity. **d**) MDA-MB-231 cell line was suspended in ALDH1 assay buffer and subjected to ALDH1 activity assay using florescence activated cytometric analysis. **e**-**f**) Control MDA-MB-231 and HCC1806 cells were stained with PE conjugated CD44 and subjected to cytometric analysis. **g**-**i**) Breast cancer cells (2x10^6^) with and without treatment with DETA NONOate (1mM) for 24h were implanted subcutaneously (ventro-lateral site) in nude mice and tumor volume was monitored for 15 weeks (*n* = 5). Data are represented as mean ± SD (**, *p* ≤ 0.01, ***, *p* ≤ 0.001, ns: non-significant)

Mammary cancer stem cells (MCSCs) are a small subset of undifferentiated cells in breast tumors that have been highly implicated in its initiation and progression [37]. Most therapeutic and chemotherapeutic drugs has been found to kill more differentiated cells while MCSCs evade these treatments [38]. Heterogeneity exists within MCSCs populations, which have high aldehyde dehydrogenase 1 (ALDH1) activity and increased expression of CD44, a cell surface marker [39]. There are reports of high expression of ALDH1 in breast tumors in women from African origin [40, 41]. We found high ALDH1 expression in all the 3 AA TN breast cancer cells examined and NO treatment significantly reduced the expression of ALDH1 in these cells (Fig. 6a). The higher expression of ALDH1 in AA TN breast cancer cells sharply contrasts with its much lower basal expression in CA TN breast cancer cells (Fig. 6a). We however, found much higher CD44 expression in the CA breast cancer cells that further increased with NO treatment (Fig. 6a). We further confirmed by Western blot that the increase in caspase cleavage in AA TN breast cancer cell lines with NO treatment occurred simultaneously with decrease in MCSC population (data not shown).

We further measured ALDH1 activity in HCC-1806 and HCC-70 after treatment with DETA-NONOate (1mM, 24-36h). We found high basal activity of ALDH1 in HCC-1806 (11.8 %) and HCC-70 (11.3 %) cells (Fig. 6b-c). Upon NO exposure there was considerable decline in ALDH1 activity in both the cell lines, HCC-1806 (6.8 %) and HCC-70 (5.6 %) (Fig. 6b-c). A large amount of dead cells were found in both the cell lines after NO treatment. We were unable to detect any ALDH1 activity in MDA-MB-231 cells (Fig. 6d). On the other hand, a large number of MDA-MB-231 cells (17.4 %) expressed CD44, which was undetected in HCC-1806 cells (Fig. 6e-f).

We further examined whether NO treatment of AA and CA breast cancer cells influenced tumor formation *in vivo*. For this purpose, we implanted control and NO-treated (for 24h) AA and CA TN breast cancer cells (2x10^6^) subcutaneously (at a ventro-lateral site) in nude mice and monitored tumor volume for 15 weeks. When compared to the untreated control cells, NO treatment of AA TN breast cancer cells dramatically reduced tumor volume in nude mice (Fig. 6b, c). These results were in sharp contrast to that observed in CA TN breast cancer cells where NO treatment led to formation of larger xenografts when compared to similar treatment groups from AA TN breast cancer cells (Fig. 6d). Our data, therefore, indicate that NO treatment of AA TN breast cancer cells significantly reduce MCSC content and xenograft formation.

## Discussion

Breast cancer in AA women usually have aggressive characteristics and unique biology. In this study, we report that nitrosative stress induced by NO, promoted apoptosis preferentially in all the AA but not in CA TN breast cancer cells. Specifically, NO treatment induced cleavage of pro-apoptotic Bax and an increase in caspase-3 activity in AA but not in CA TN breast cancer cells. Interestingly, a decline in anti-apoptotic Bcl2 and depolarization of MMP was observed in AA breast cancer cells. In sharp contrast, Bcl2 levels remained steady or even increased along with hyperpolarization of MMP in NO treated breast cancer cells of CA origin. There was an early induction of HO-1, a stress response gene, in AA breast cancer cells, which were sensitive to nitrosative stress. Levels of HO-1 did not change in any of the CA cells with NO exposure, further indicating their insensitivity to nitrosative stress. In another study, NO treatment was found to induce higher increase migration and invasiveness of MDA-MB-231 (CA TN origin) when compared to MDA-MB-468 (AA TN origin) breast cancer cells [42]. It appears that NO elicits tumor promoting responses in CA breast cancer cells that sharply contrasts to the death-promoting signals in AA cells. We have previously shown that low concentrations of NO in addition to increasing proliferation of MDA-MB-231 cells, activated mammalian target of rapamycin (mTOR) to increase translation of proteins [18]. Studies also show that ROS/RNS drive a continuous process of DNA adducts that crosslinks as well as promoting posttranslational modification of lipids and proteins that increase survival, immunosuppression and inhibition of apoptosis [43]. These could be some of the mechanisms by which NO influenced tumor promoting behavior in CA breast cancer cells.

In our study, the basal levels of ROS were comparable in breast cancer cells from the two ethnic populations. However, NO treatment specifically increased ROS only in AA TN breast cancer cells. This increase in ROS coincided with reduction in the activity of total (mitochondrial and cytosolic) SOD in AA TN breast cancer cells. Our study further shows that there was no significant difference in the stability of SOD in AA and CA TN breast cancer cells. There are reports of reduced SOD activity associated with increased nitrosative stress in AAs but not in CA umbilical vein endothelial cells (HUVEC) [44]. Inverse relationship between NO and SOD activity was also evident in plasma of AA patients suffering from hypertension [45]. Older AA as well as those that underwent spontaneous preterm birth had lower SOD activity when compared to similar group of CA patients [46]. No difference in Mn-SOD Ala-9Val polymorphism in breast tumors from AA and CA patients has been detected [47]. However, a striking difference in the frequency of 10T/9T intron 3 polymorphism in mitochondrial SOD in AAs has been reported [48]. Although sensitivity of AA population to NO-mediated inactivation of SOD is well demonstrated, molecular mechanisms and consequences of SOD inactivation remain poorly understood.

Prominent differences in the biology of AA and CA breast tumors suggest differences in both the tumor cells and the microenvironment in which these tumors develop. Despite significant differences in biology there is not much difference in the treatment strategies for these tumors. Tumor infiltrating macrophages in poorly differentiated breast carcinoma, express active iNOS that promote angiogenesis, increase tumor size and cause poor survival of patients [16]. More recently, targeting endogenous iNOS in two CA breast cancer cell lines MDA-MB-231 and BT-549, by selective iNOS and pan-NOS inhibitors, was found effective in reducing tumor volume in mouse model [49].

Our previous studies indicate AA TN breast cancer cells utilize arginine preferentially to synthesize polyamines [21, 22]. Arginase and nitric oxide synthase (NOS) compete for cellular arginine to produce polyamines and NO respectively [21, 22]. Elevated arginase activity in cells has been found to both decrease cellular availability of NO by competing with NOS to increase L-ornithine. We have shown that arginase expression is up-regulated in AA breast cancer cells, which are dependent on polyamines for survival and proliferation [21]. Therefore exploiting ethnic differences in arginine metabolism has the potential to provide selective druggable targets that could effectively reduce aggressiveness of CA and AA TN breast tumors. Although there is ample evidence of unique biology of AA TN breast tumors, no potential druggable target has so far been identified or exploited to reduce the severity of the disease*.*

The potential of using NO to induce apoptosis in AA breast tumors appears promising for therapeutic intervention. Depending on the cell type, NO has shown promise as a therapeutic agent to reduce tumor volume [50, 51]. The therapeutic potential of NO-donors depends on its capacity to release NO at optimum concentrations and in a temporally regulated manner to kill tumor cells. A number of potential NO-donors include organic nitrites glyceryl trinitrite (GTN), metal-nitrosyl complexes sodium nitroprusside (SNP), S-nitrosoglutathione (GSNO) and diazeniumdiolates. Studies have shown that GTN induces apoptosis in colon cancer, inhibit hypoxia-mediated metastatic potential of B16F10 murine melanoma cells and increase the chemosensitivity of human prostate tumor xenografts [52–54]. Diethylene diazeniumdiolates (NONOates) has been found to be an effective chemo preventive agent against bone metastatic breast cancer as well as aggressive breast cancer cell lines [55]. Unfortunately, many of these NO-donors lack target specification and controlled release kinetics, therefore cannot be tested for their efficacy *in vivo*. Considering dual effects of NO and lack of ideal NO-donors, we are not equipped technically to use NO for therapy.

## Conclusions

AA and CA TN breast cancer cells respond differently to nitrosative stress suggesting differences in their biology. Increased sensitivity of AA TN breast tumors to nitrosative stress-mediated apoptosis suggest exploring the potential of NO as a therapeutic agent. Therapeutic strategies should be tailored to the unique biology of the disease.

## Abbreviations

AA, African American; CA, Caucasian; TN, Triple negative; NO, Nitric Oxide; SOD, Superoxide dismutase; ROS, Reactive oxygen species; ALDH1, Aldehyde dehydrogenase1; MCSCs, Mammary cancer stem cells; ER, Estrogen receptor; PR, Progesterone receptor; NOS, Nitric oxide synthase; RNS, Reactive nitrosative stress; MKP-1, MAP kinase phosphatase-1; TUNEL, Terminal deoxynucleotidyl transferase dUTP nick end labeling; HO-1, Hemeoxygenase1; MMP, Mitochondrial membrane potential; PT, Mitochondria permeability transition; NAC, N –acetyl cysteine
